# Hip Impingement after Anterior Inferior Iliac Spine Avulsion Fractures: A Case Report with Review of the Literature

**DOI:** 10.1155/2020/8893062

**Published:** 2020-10-20

**Authors:** Mark J. Lambrechts, Aaron D. Gray, Dan G. Hoernschemeyer, Sumit Kumar Gupta

**Affiliations:** University of Missouri Department of Orthopaedic Surgery, Columbia, MO, USA

## Abstract

Avulsion fractures of the anterior inferior iliac spine (AIIS) are rare injuries in adolescent athletes. We present a case of a 15-year-old male who sustained an avulsion injury to his right AIIS when kicking a soccer ball. The patient had chronic pain and extra-articular subspinal impingement leading to decreased hip flexion and rotation. The injury occurred 1.5 years prior to symptom onset, and we were the first health care providers to manage the injury. We attempted six months of nonoperative management including activity modifications and nonsteroidal anti-inflammatory (NSAID) therapy without improvement. Although this injury can often be managed nonoperatively, his symptoms required excision of the AIIS and associated heterotopic ossification. He had an excellent outcome with return to soccer and no pain at his final follow-up visit two years after surgery. Due to the limited literature guiding the surgeon's management of AIIS avulsion injuries with associated heterotopic ossification, we provide a review of the literature detailing pre- and postoperative ranges of motion, surgical approach, fixation or excision of the avulsion fragment, and return to sport in this patient population.

## 1. Introduction

Avulsion fractures of the adolescent pelvis are a well-known phenomenon, but the incidence is rare. High-level adolescent soccer players, track and field athletes, gymnasts, and tennis players are known to be at high risk for these injuries [1]. The site of avulsion also appears to be dependent on the particular sport being played. Soccer players are at high risk of avulsion fractures of the anterior inferior iliac spine (AIIS) due to the forceful contracture of the rectus femoris during hip flexion and knee extension when kicking. In fact, approximately 50% of AIIS avulsion fractures are due to kicking [2]. These fractures can be difficult to diagnose since they are often not visible on standard pelvis X-rays, and a high degree of clinical suspicion is required. Although previous case reports have demonstrated AIIS avulsion fractures months to years after the injury with excessive callus formation and minor decreases in range of motion, other patients have severely limited motion [3, 4]. A combination of the failure to diagnose an acute pelvic avulsion fracture with X-ray and the acquired heterotopic ossification, which can mimic other more dire diagnoses, makes an initial diagnosis of the pelvic avulsion fracture integral in properly treating these patients [5].

The patient and his parents gave informed consent to publish this case report.

## 2. Case Report

A 15-year-old male presented to the clinic with a complaint of right hip pain and decreased range of motion of his hip. A detailed history was performed, which included the patient recalling an injury 18 months prior when he was playing in a nonorganized soccer match. He felt a muscle pull in his right groin. He took a break from playing but resumed after a 10-minute rest. Due to continued pain he eventually stopped playing for the day. Over the next year, he noticed an increased difficulty with hip flexion especially with squatting and kicking a soccer ball with an associated increase in pain. He presented to our clinic for evaluation 18 months after the initial injury. We were the first doctors to see him regarding his right hip injury.

On physical examination, there was a large hard mass in the area of his right groin. His hip flexion was limited to 70 degrees on the injured extremity compared to 120 degrees on the contralateral side. He had 0 degrees of external rotation. However, he still had 40 degrees of internal rotation.

**Figure 1:**
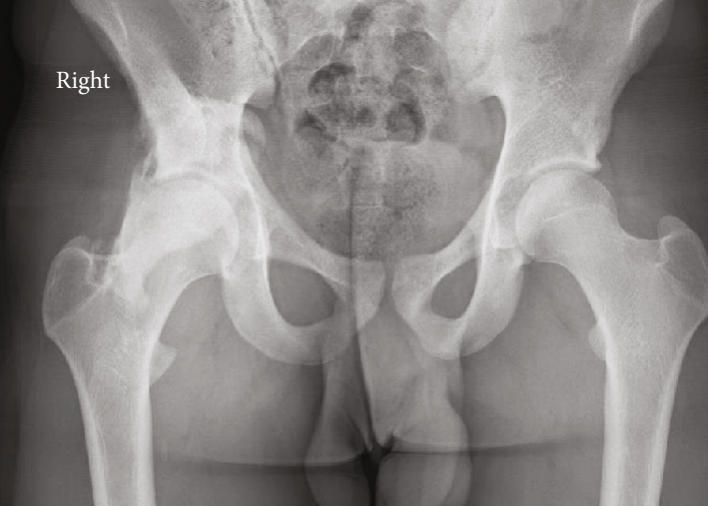
AP X-ray of the pelvis demonstrating an avulsion fracture of the right AIIS with large amount of bony callus.

**Figure 2:**
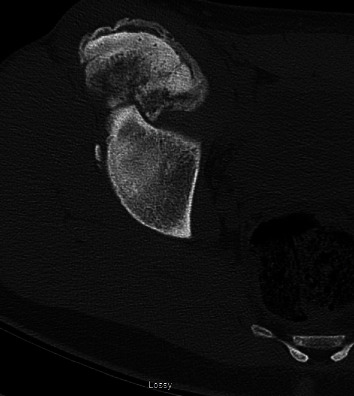
Axial CT scan demonstrating the large amount of bone deposit from the right AIIS avulsion fracture.

**Figure 3:**
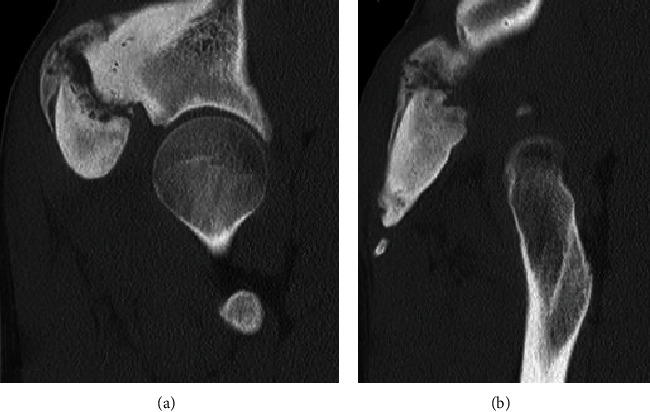
(a) Coronal CT image demonstrating the right AIIS and the large amount of heterotopic ossification. (b) Another sequence from the coronal CT imaging demonstrating the extent of bone growth.

Imaging with X-rays was remarkable for a large heterotopic bone mass at the AIIS that extended 117 mm distal to the AIIS and a maximum lateral distance of 64 mm (Figure 1). Computed tomography (CT) imaging was obtained, which confirmed our suspicion of the diagnosis of an AIIS avulsion fracture with a 117 mm × 65 mm bony “mass” (Figures 2 and 3). At this time, his diagnosis was consistent with extra-articular subspinal impingement. The patient failed 6 months of conservative management including physical therapy, activity modification, and nonsteroidal anti-inflammatory drugs (NSAIDs). Therefore, surgical treatment for excision of the mass was offered.

**Figure 4:**
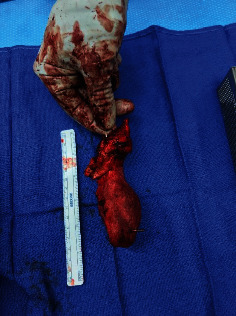
Picture demonstrating actual size (117 mm × 65 mm) of the heterotopic ossification.

**Figure 5:**
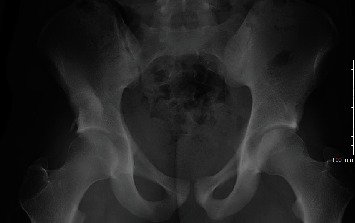
Postoperative X-ray 1 year after heterotopic bone removal.

We proceeded to the operating theater and removed the heterotopic ossification through an anterior Smith-Peterson approach to the hip (Figure 4). We found that a large part of the origination of the rectus femoris was still attached to the distal aspect of the mass. This was detached and tenodesed. Postoperatively, the patient was placed on a course of indomethacin for 1 month. He returned to playing soccer after his two-month follow-up visit. The postoperative X-rays at one-year follow-up demonstrated no new bone deposition (Figure 5). He had 120-degree hip flexion and full internal and external ranges of motion at his two-year follow-up visit. Importantly, the patient rated his pain at 0/10 at his 2-month, 12-month, and 24-month postoperative visits.

## 3. Discussion

Avulsion fractures of the pelvis are often due to an atraumatic event during sports [6]. Sprinting and kicking are often the inciting events due to forceful contractions of the rectus femoris and hamstring muscles, respectively. Avulsion fractures often occur in adolescents due to the apophysis being the biomechanical weak link in the tendon-bone kinetic chain [7]. In adults, these forceful contractions lead to muscle strains or tears since the apophysis has closed.

The management of these injuries has been predominantly nonoperative. Return to sports with nonoperative treatment is usually within 2 to 3 months when following the protocol of Metzmaker and Pappas [8]. This includes rest, ice, and NSAIDs for the first week, gentle passive range of motion and assisted weight bearing during week two, resistance training in the third and fourth weeks, and sports-specific training and exercises 4 to 8 weeks after injury. After 2 months, return to sport is usually allowed. However, AIIS fractures are at increased risk of pain with those greater than 2 cm displaced also at greater risk of nonunion [9]. Large amounts of heterotopic ossification causing symptomatic hip impingement and unremitting pain indicate failures of nonoperative treatment and require operative intervention [10].

Currently, there is some debate whether operative or nonoperative treatment of acute AIIS avulsion fractures has equivalent outcomes. Generally, close follow-up with nonoperative management is an appropriate treatment choice. However, a recent meta-analysis of 596 patients has shown superior outcomes with operative management. Return to sport was 92% in those treated operatively versus 80% in nonoperatively treated patients, and those with fractures displaced greater than 15 mm and elite-level athletes should be considered relative indications to forgo a trial of nonoperative treatment [11].

We performed a literature review to identify all patients requiring surgical intervention of an AIIS avulsion fracture treated nonoperatively with associated heterotopic ossification. Only articles in the English language were considered for inclusion. The following MeSH words were linked “anterior inferior iliac spine” OR “AIIS” AND “avulsion.” We identified 8 manuscripts accounting for 9 cases of failed conservative management due to femoroacetabular impingement, pain with hip flexion, or subspinal impingement and reviewed the type of surgical approach, time to return to sport, and improvement in range of motion [4, 10, 12–17]. We believe the following questions are relevant for guiding future research: (1) Is arthroscopic or open excision of heterotopic ossification superior, (2) Is fixation of the fragment superior to excision, (3) Is there an improvement in range of hip motion after surgery, and (4) Do symptoms leading to surgical treatment affect patient outcomes (Table 1)? Due to the limited number of cases reported in the literature, none of these questions can be answered using evidence-based practice at this time. Additionally, large case series including preoperative and postoperative range of motion, reason for operative intervention, type of surgical approach, and time to return to sport should be documented for each reported case to improve treatment in this cohort of patients.

We present this case to highlight the importance of identifying atraumatic injuries of the pelvis in adolescent athletes. If there is suspicion of a pelvic avulsion fracture after physical exam, X-rays should be obtained and a follow-up visit should be ordered. Although an initial clinic visit should be within a month to determine if the patient is improving, it would be reasonable to set a second visit at 4 months as heterotopic ossification is almost universally identified by this time [18]. Parents can be informed that they may call to cancel the appointment if the patient is asymptomatic and back to sporting events. If the pelvic injury is chronic and the patient history and X-ray appear to be inconsistent with a history of an avulsion fracture, a CT scan is the preferred next test. This will identify heterotopic ossification and exclude unlikely differential diagnoses [19].

## 4. Conclusion

Adolescent anterior iliac spine fractures are uncommon injuries during sporting events caused by the apophysis being the weak point of the muscle-tendon junction. AIIS avulsion fractures can be managed nonoperatively if the fracture has minimal displacement and the patient plays sports recreationally. These injuries can cause significant functional limitations and pain. This necessitates operative management usually resulting in good outcomes.

## Figures and Tables

**Table 1:** List of publications of anterior inferior iliac spine avulsion fractures treated nonoperatively with subsequent development and surgical excision of heterotopic ossification at the site of fracture.

Authors	Diagnosis	ROM prior to surgery/after surgery	ORIF of AIIS fragment?	Approach	Return to sport	Follow-up/recurrence
Alhaneedi et al. [4]	Femoroacetabular impingement	N/A	No	Open	N/A	2 years
Larson et al. [10]	Femoroacetabular impingement	110 degrees flexion/125 degrees flexion	No	Arthroscopic	N/A	1.5 years
Nakano et al. [12]	Extra articular subspine impingement	N/A	No	Arthroscopic	N/A	1 year/none
Carr et al. [13]	Extra articular subspine impingement	N/A	Yes	Open	5 months	1 year/none
Novais et al. (case 1) [14]	Extra articular subspine impingement	90 degrees flexion, 10 degrees internal rotation/100 degrees and 30 degrees	No	Open	6 months	26 months
Novais et al. (case 2) [14]	Extra articular subspine impingement	100 degrees flexion/120 degrees flexion and 35 degrees internal rotation	No	Open	6 months	28 months
Shibahara et al. [15]	Extra articular subspine impingement	90 degrees flexion, 20 degrees internal rotation, 30 degrees external rotation	No	Arthroscopic	4 months	N/A
Rajasekhar et al. (case 1) [16]	Hip pain with flexion	“Within normal limits”	Yes	Open	N/A	2.5 years
Matsuda and Calipusan [17]	Femoroacetabular impingement	N/A	No	Arthroscopic	N/A	18 months
